# Supplementary material for: From birth to lying on- or under a supplemental heat source: How long does it take for half the litter to lie down and how long do they stay?
Source: PLoS One. 2023 Dec 21;18(12):e0294336. doi: 10.1371/journal.pone.0294336 (PMC10734949; doi:10.1371/journal.pone.0294336)
Supplement: Attachment — Submitted filename: Response to Reviewers.docx [file pone.0294336.s001.docx]

IOWA STATE UNIVERSITY

College of Agriculture

Department of Animal Science

Animal Physiology Section

2356-F Kildee Hall

Ames, Iowa 50011-3150

Phone: 515-294-2098

FAX: 515-294-4471

E-mail: johnsona@iastate.edu

OF SCIENCE AND TECHNOLOGY

Below you will find our responses to the reviewers and editor’s directions.

Please include the following on our behalf per your direction below:

**Funding and the role of the funder:**

This project was funded in part by Iowa Farm Bureau Federation (AJ) and Kane Manufacturing (KS). Partial funding of Dr. Johnson’s and Dr. Stalder’s salary is supported by the Department of Animal Science, College of Agriculture and Life Sciences at Iowa State University, and the US Department of Agriculture. The funders had no role in study design, data collection and analysis, decision to publish or preparation of the manuscript.

As it relates to data, we note that you have indicated that data from this study are available upon request. PLOS only allows data to be available upon request if there are legal or ethical restrictions on sharing data publicly. For more information on unacceptable data access restrictions, please see <http://journals.plos.org/plosone/s/data-availability#loc-unacceptable-data-access-restrictions>.

AU: Please use the IRB at ISU for a non-author contact:

Data availability:

**Dr. Raeann Ritland, IRB Post-Approval Monitor**
[**(515) 294-7792**](tel:%20+15152947792)
[**raeannr@iastate.edu**](mailto:raeannr@iastate.edu)

I will need some guidance on this. Dr. Kenneth Stalder died last October (2022) and therefore, I cannot garner his approval on allowing the data to be publicly shared. Being the corresponding author and mentor for Dr. Stambuk can I make this decision for the team?

If we can be of further help, please do not hesitate to contact us.

Sincerely,

Anna Johnson, Ph.D. Associate Professor Animal Behavior and Welfare

PONE-D-23-21093

From Birth to Lying on- or Under a Supplemental Heat Source: How Long does it take for Half the Litter to Lie Down and How Long Do They Stay?

PLOS ONE

Dear Dr. Johnson,

Thank you for submitting your manuscript to PLOS ONE. After careful consideration, we feel that it has merit but does not fully meet PLOS ONE’s publication criteria as it currently stands. Therefore, we invite you to submit a revised version of the manuscript that addresses the points raised during the review process.

Please submit your revised manuscript by Oct 07 2023 11:59PM. If you will need more time than this to complete your revisions, please reply to this message or contact the journal office at [plosone@plos.org](mailto:plosone@plos.org). When you're ready to submit your revision, log on to <https://www.editorialmanager.com/pone/> and select the 'Submissions Needing Revision' folder to locate your manuscript file.

Please include the following items when submitting your revised manuscript:

- A rebuttal letter that responds to each point raised by the academic editor and reviewer(s). You should upload this letter as a separate file labeled 'Response to Reviewers'.
- A marked-up copy of your manuscript that highlights changes made to the original version. You should upload this as a separate file labeled 'Revised Manuscript with Track Changes'.
- An unmarked version of your revised paper without tracked changes. You should upload this as a separate file labeled 'Manuscript'.

If you would like to make changes to your financial disclosure, please include your updated statement in your cover letter. Guidelines for resubmitting your figure files are available below the reviewer comments at the end of this letter.

If applicable, we recommend that you deposit your laboratory protocols in protocols.io to enhance the reproducibility of your results. Protocols.io assigns your protocol its own identifier (DOI) so that it can be cited independently in the future.

For instructions see: <https://journals.plos.org/plosone/s/submission-guidelines#loc-laboratory-protocols>. Additionally, PLOS ONE offers an option for publishing peer-reviewed Lab Protocol articles, which describe protocols hosted on protocols.io. Read more information on sharing protocols at <https://plos.org/protocols?utm_medium=editorial-email&utm_source=authorletters&utm_campaign=protocols>.

We look forward to receiving your revised manuscript.

Kind regards,

Ewa Tomaszewska, DVM Ph.D
Academic Editor
PLOS ONE

Journal requirements:

When submitting your revision, we need you to address these additional requirements.

1. Please ensure that your manuscript meets PLOS ONE's style requirements, including those for file naming. The PLOS ONE style templates can be found at

<https://journals.plos.org/plosone/s/file?id=wjVg/PLOSOne_formatting_sample_main_body.pdf> and

<https://journals.plos.org/plosone/s/file?id=ba62/PLOSOne_formatting_sample_title_authors_affiliations.pdf>

**AU: Thank you for these helpful links. The documents were referred to in the updating of the manuscript.**

2. To comply with PLOS ONE submissions requirements, in your Methods section, please provide additional information regarding the experiments involving animals and ensure you have included details on (1) methods of sacrifice, (2) methods of anesthesia and/or analgesia, and (3) efforts to alleviate suffering.

**AU: Added the following ethical statement “Sows or piglets were all cared for following the approved farms standard operating procedures. No sows or piglets were anaesthetized, provided analgesics or euthanized during the study.”**

3. Thank you for stating the following financial disclosure:

“This project was funded in part by Iowa Farm Bureau Federation (AJ) and Kane Manufacturing (KS). Partial funding of Dr. Johnson’s and Dr. Stalder’s salary is supported by the Department of Animal Science, College of Agriculture and Life Sciences at Iowa State University, and the US Department of Agriculture.”

Please state what role the funders took in the study. If the funders had no role, please state: "The funders had no role in study design, data collection and analysis, decision to publish, or preparation of the manuscript."

If this statement is not correct you must amend it as needed.

**AU: I have added "The funders had no role in study design, data collection and analysis, decision to publish, or preparation of the manuscript."**

**DONE**

Please include this amended Role of Funder statement in your cover letter; we will change the online submission form on your behalf.

**AU: thank you**

4. Thank you for stating the following in the Acknowledgments Section of your manuscript:

“This project was funded in part by Iowa Farm Bureau Federation and Kane Manufacturing. Partial funding of Dr. Johnson’s and Dr. Stalder’s salary is supported by the Department of Animal Science, College of Agriculture and Life Sciences at Iowa State University, and the US Department of Agriculture.”

We note that you have provided funding information that is currently declared in your Funding Statement. However, funding information should not appear in the Acknowledgments section or other areas of your manuscript. We will only publish funding information present in the Funding Statement section of the online submission form.

**AU: Removed thank you**

Please remove any funding-related text from the manuscript and let us know how you would like to update your Funding Statement. Currently, your Funding Statement reads as follows:

“This project was funded in part by Iowa Farm Bureau Federation (AJ) and Kane Manufacturing (KS). Partial funding of Dr. Johnson’s and Dr. Stalder’s salary is supported by the Department of Animal Science, College of Agriculture and Life Sciences at Iowa State University, and the US Department of Agriculture.”

Please include your amended statements within your cover letter; we will change the online submission form on your behalf.

**AU: Thank you**

5. We note that you have indicated that data from this study are available upon request. PLOS only allows data to be available upon request if there are legal or ethical restrictions on sharing data publicly. For more information on unacceptable data access restrictions, please see <http://journals.plos.org/plosone/s/data-availability#loc-unacceptable-data-access-restrictions>.

**AU: I will need some guidance on this. Dr. Kenneth Stalder died last October (2022) and therefore, I cannot garner his approval on allowing the data to be publicly shared.**

In your revised cover letter, please address the following prompts:

a) If there are ethical or legal restrictions on sharing a de-identified data set, please explain them in detail (e.g., data contain potentially sensitive information, data are owned by a third-party organization, etc.) and who has imposed them (e.g., an ethics committee). Please also provide contact information for a data access committee, ethics committee, or other institutional body to which data requests may be sent.

**No ethical or legal restrictions on sharing the data set.**

**Contact for the data can be sent to** [**johnsona@iastate.edu**](mailto:johnsona@iastate.edu)

b) If there are no restrictions, please upload the minimal anonymized data set necessary to replicate your study findings as either Supporting Information files or to a stable, public repository and provide us with the relevant URLs, DOIs, or accession numbers. For a list of acceptable repositories, please see <http://journals.plos.org/plosone/s/data-availability#loc-recommended-repositories>.

**AU: Per Lisa Burna-Asefi direction, I take full responsibility for the data integrity, collection, quality, analysis and interpretation of the results on behalf of Dr. K. Stalder. You may go ahead and use my e-mail** [**johnsona@iastate.edu**](mailto:johnsona@iastate.edu) **in place of Dr. Stalder’s e-mail. Thank you.**

We will update your Data Availability statement on your behalf to reflect the information you provide.

**AU: Thank you**

6. Please include a separate caption for each figure in your manuscript.

**AU: Per Lisa Burna-Asefi direction “place the figure headings in the location of the main manuscript, but the figures are placed at the end of the manuscript.” DONE**

7. Please review your reference list to ensure that it is complete and correct. If you have cited papers that have been retracted, please include the rationale for doing so in the manuscript text, or remove these references and replace them with relevant current references. Any changes to the reference list should be mentioned in the rebuttal letter that accompanies your revised manuscript. If you need to cite a retracted article, indicate the article’s retracted status in the References list and also include a citation and full reference for the retraction notice.
[Note: HTML markup is below. Please do not edit.]
**AU: DONE AND CORRECT**

Reviewers' comments:

Reviewer's Responses to Questions

**Comments to the Author**

1. Is the manuscript technically sound, and do the data support the conclusions?

The manuscript must describe a technically sound piece of scientific research with data that supports the conclusions. Experiments must have been conducted rigorously, with appropriate controls, replication, and sample sizes. The conclusions must be drawn appropriately based on the data presented.

Reviewer #1: Yes

2. Has the statistical analysis been performed appropriately and rigorously?

Reviewer #1: N/A

3. Have the authors made all data underlying the findings in their manuscript fully available?

The [PLOS Data policy](http://www.plosone.org/static/policies.action#sharing) requires authors to make all data underlying the findings described in their manuscript fully available without restriction, with rare exception (please refer to the Data Availability Statement in the manuscript PDF file). The data should be provided as part of the manuscript or its supporting information, or deposited to a public repository. For example, in addition to summary statistics, the data points behind means, medians and variance measures should be available. If there are restrictions on publicly sharing data—e.g. participant privacy or use of data from a third party—those must be specified.

Reviewer #1: Yes

4. Is the manuscript presented in an intelligible fashion and written in standard English?

PLOS ONE does not copyedit accepted manuscripts, so the language in submitted articles must be clear, correct, and unambiguous. Any typographical or grammatical errors should be corrected at revision, so please note any specific errors here.

Reviewer #1: Yes

5. Review Comments to the Author

Please use the space provided to explain your answers to the questions above. You may also include additional comments for the author, including concerns about dual publication, research ethics, or publication ethics. (Please upload your review as an attachment if it exceeds 20,000 characters)

Reviewer #1: This is a very nice study looking at a very simple practice that can lead to improved caretaker tools and observation practices. Some comments are provided below to clarify information about the purpose and background of the goals of this manuscript.

**AU: Thank you**

Line 54: Remove the dash after 250. In the second sentence of line 54, please remove “, respectively”.

**AU: DONE**

Lines 59-61: Curious as to why the authors were only interested in looking at half the litter as the minimum point. Can the rationale be explained why at least half the litter was the target for this research? Why not the full litter? Or 75% or some other proportion?

**AU: Dr. Stalder was the one who crafted this idea. Of course, we can not double check this with Dr. Stalder but from our notes and recollections, he decided that more than half the litter was a good indicator of performing a behavior because it’s a majority, especially when we couldn’t count piglets individually (as they were not ID).**

Line 70: Remove the comma after “Location”

**AU: DONE**

Line 76: Please rephrase: "...creep area, where the heat source provided measured 1.2 m..."

**AU: DONE**

Line 78: Remove the parenthesis at end of reference.

**AU: DONE**

Line 116, last sentence: Why were no statistical analyses completed or presented in this manuscript

**AU: The team did try to analysis with the data set. The model and details are as follows:**

To facilitate analysis and interpretation, sows were grouped by their parity into 3 groups (group 1 included parity 1 sows, group 2 included parities 2 to 4 sows, and group 3 included parities ≥ 5 sows). Number born was defined as the number of piglets born alive after 24 h. All data was evaluated using mixed model methodology (Proc Mixed, SAS version 9.4, SAS Institute Inc., Cary, NC). Fixed effects in the model included treatment, group, room, and number born. A random effect for farrowing batch was included in the analysis models. Sources of model variation were considered significant at *P* < 0.05. Least squares means for each level within treatment were separated using the pdiff option within the Proc Mixed procedure. All analyses were conducted where the unit of measure was seconds (s) and converted to minutes (min).

Results were as follows:

No treatment differences were observed in the latency (*P* = 0.30) or duration (*P* = 0.26) of piglet first heat source use when comparing heat lamps versus heat mats. The LSM ± SE for latency of first heat source use after birth was 197 ± 185.7 min (11,822 ± 11,139 s; approximately 3 h) for LAMP and 403 ± 183.0 min (24,170 ± 10,979 s; approximately 7 h) for MAT while the duration of first heat source use was 32 ± 6.1 min (1,920 ± 363.6 s) for LAMP and 42 ± 5.6 min (2,536 ± 333.2 s) for MAT.

**However, when you look closely at the data set there were real concerns with the stats (see that the SE is almost as large as the LSMeans). Therefore, upon statistical advise (KJS) we decided that the data set was too small to have the ability to conduct correct and powerful stats and therefore we moved away from this and to a simple yet effective descriptive only approach.**

Line 119-120: Was parity evenly distributed between treatments?

**AU: Yes, we did. The process was as follows, the study group was a small cohort of sows at the University farm (n = 12). We then took the 12 and equally divided this into the two trt groups. Then, we tried as best we could to balance the parity structure within this. Previous work by Stalder notes a P3+ sow is more productive and makes the money. So, from there we balanced the parity by trt as follows:**

| **TRT** | **Parity (1 to 2)** | **3+** |
| --- | --- | --- |
| LAMP | 2 | 4 |
| MAT | 2 | 4 |

Line 127: Please rephrase as: "When breaking down the first lying event by sow parity and heat source,..."

**AU: DONE**

Line 128: where it is stated “MAT piglets”, please remove the word piglets here...it is already stated earlier in the sentence.

**AU: DONE**

Lines 137-140: Please provide more discussion on how newborn piglets perceive their environment, particularly to seeking warmth and needs for thermoregulation.

**AU: The following has been added:**

**Pigs are attracted to their dam’s udder immediately following birth [19]. This attraction to the udder is driven by olfactory cues with piglets having a highly developed sense of smell [20, 21]. (Morrow-Tesch and McGlone, 1990; Rohde Parfet and Gonyou, 1991). Other attractors towards the sow udder include both tactile and thermal properties [19, 22].**

Lines 140-142: Please provide more context on the lack of ABM for caretakers or currently used ABM that are practical and used in commercial swine settings. Please emphasize why its important for ABMs to be practical and what is needed to provide more tools like these to caretakers.

**AU: I have added information to address this in the below comments. Is this sufficient? If not, let me know and I can flesh this out further.**

Lines 142-143: This is the first mention of this study focusing on its goals relative to "a new practical ABM". This should be introduced in the intro section and more detail should be provided here to emphasize how this study's methods yield such an ABM that would be practical at the farm/caretaker-level.

**AU: Great suggestion. We have added in a new paragraph in the introduction to “set this idea up” for the reader. We have added the following:**

**Training caretakers with practical Animal Based Measures (ABM) is critical for neonatal management to improve survivability. These ABM’s may include knowing the time interval between piglet births and determining if assistance is required to drying piglets. What would be valuable is to design and test a novel on-farm practical ABM to improve neonatal survivability related to the important heat source in the farrowing area (*personnel communication*). If caretakers can determine how quickly, or not piglets seek and use the heat source, they can play a more proactive role in helping some piglets seek warmth and, in turn improve the chances of survival.**

**In the discussion we added the following:**

**It should be noted, that this ABM was not designed to determine the first- and length of time an individual piglet “first” used the heat source rather when ≥ 50% of piglets to lie on- or under the supplemental heat source for ≥ 5 min after the birth of the last-born pig. This 50% was a starting point, as the team considered this a “majority.” However, future work could consider other increments and relate this back to survival, such as 25, 75 or 100% respectively.**

**This ABM fits in to today’s protocols and practices for the newborn piglet. Caretakers on commercial farms do not identify and track the piglet at this early stage of life on an individual level. Thus, creating a cheap, effective and practical on-farm tool could aid caretakers in better time management to assist newborns who are struggling to locate the heat source. Knowing when a sow ends farrowing would be the “starting point” for caretakers to note on the sow card, and before they leave for the day they can make sure on their last walk through that at ≥ 50% of piglets to lie on- or under the supplemental heat source for ≥ 5 min after the birth of the last-born pig. If they locate litters that have not met this ABM then they can duly move piglets onto- or under the heat source and make an additional note on the sow card.**

**We hope this is what the reviewer was thinking – if not please let us know and we will refine further.**

Line 143: Please begin sentence with "On the farm".

**AU: DONE**

Lines 144-147: Please rephrase this last section of the sentence. It is difficult to follow.

**AU: Done – with the addition above I think this will help but I also reframed it a bit:**

**It should be noted, that this ABM was not designed to determine the first- and length of time an individual piglet “first” used the heat source. Rather it was designed for a caretaker to be able to determine when ≥ 50% of piglets to lie on- or under the supplemental heat source for ≥ 5 min after the birth of the last-born pig. This 50% was a starting point, as the team considered this a “majority.” However, future work could consider other increments and relate this back to survival, such as 25, 75 or 100% respectively.**

Lines 150-153: Do the authors believe that although it took piglets longer to find the mats, this may be why they chose to stay on the mat longer?

**AU: Davis and others [13] reported that mats provide a more evenly distributed surface temperature and are considered bottom-up heating with their function to heat piglets through their stomachs, which may increase the desire to lie down. So, it would seem like a reasonable hypothesis. I think some preference testing in this space is also needed.**

Line 154: Remove comma after "seeking"

**AU: DONE**

Line 165: Please move the comma to after the reference.

**AU: DONE**

Lines 166-168: Are there any studies where preference testing of heat sources by piglets was conducted?

**AU: Good question. I dug back into the literature and I came across two studies that showed promise BUT neither of them sets it up as a preference study. The heating trt was allocated one to each stall. Hence, piglets could not choose which one to use.**

[Does light attract piglets to the creep area?](https://pubmed.ncbi.nlm.nih.gov/25711807/)

Larsen ML, Pedersen LJ. Animal. 2015 Jun;9(6):1032-7. doi: 10.1017/S1751731115000257.

Tierarztl Prax Ausg G Grosstiere Nutztiere 2015;43(4):207-15. doi: 10.15653/TPG-150068. Epub 2015 Jul 13.

# Comparison of novel infrared heating plates and conventional warm water plates for piglets' creep areas in farrowing pens

[L Strauch-Sürken](https://pubmed.ncbi.nlm.nih.gov/?term=Strauch-S%C3%BCrken+L&cauthor_id=26166364), [M Wendt](https://pubmed.ncbi.nlm.nih.gov/?term=Wendt+M&cauthor_id=26166364)

Line 166: Check formatting for references, see a bracket and parenthesis here.

**AU: DONE THE ) WAS REMOVED**

Line 175: Please rephrase as: "should be trained to use this ABM..."

**AU: DONE**

Table 1, line 252 (Footnote): "...born alive IN litters that..." Missing word "in"

**AU: DONE**

Table 2: Please include a header/title for the last column of the table.

**AU: DONE added “Energy”**

Figure 1, line 2 (Figure description): Add the word "for" after farrowing. "...after farrowing FOR ..."

**AU: DONE**

Figure 1, line 4 (Figure description): Add parenthesis at end of reference.

**AU: DONE**

Figure 2 graph: Please relabel your x-axis as "Time (h:min)"

**AU: DONE**

Figure 3 graph: Please center your axis label below the treatments and relabel as "Time (min)"

**AU: DONE**

6. PLOS authors have the option to publish the peer review history of their article ([what does this mean?](https://journals.plos.org/plosone/s/editorial-and-peer-review-process#loc-peer-review-history)). If published, this will include your full peer review and any attached files.


If you choose “no”, your identity will remain anonymous but your review may still be made public.


**Do you want your identity to be public for this peer review?** For information about this choice, including consent withdrawal, please see our [Privacy Policy](https://www.plos.org/privacy-policy).

Reviewer #1: **Yes:** Michelle Calvo-Lorenzo

[NOTE: If reviewer comments were submitted as an attachment file, they will be attached to this email and accessible via the submission site. Please log into your account, locate the manuscript record, and check for the action link "View Attachments". If this link does not appear, there are no attachment files.]

While revising your submission, please upload your figure files to the Preflight Analysis and Conversion Engine (PACE) digital diagnostic tool, <https://pacev2.apexcovantage.com/>. PACE helps ensure that figures meet PLOS requirements. To use PACE, you must first register as a user. Registration is free. Then, login and navigate to the UPLOAD tab, where you will find detailed instructions on how to use the tool. If you encounter any issues or have any questions when using PACE, please email PLOS at [figures@plos.org](mailto:figures@plos.org). Please note that Supporting Information files do not need this step.

*In compliance with data protection regulations, you may request that we remove your personal registration details at any time.* [*(Remove my information/details)*](https://www.editorialmanager.com/pone/login.asp?a=r)*. Please contact the publication office if you have any questions.*
